# Case Report: clinical manifestations and imaging features associated with PANK2 c.940C>T variant in PKAN with symmetric basal ganglia calcification

**DOI:** 10.3389/fgene.2026.1792232

**Published:** 2026-06-10

**Authors:** Feng Liang, Rui Ban, Jiajun Li, Jian Li, Shanglin Li, Jia Chen, Di Wang, Yanan Liu, Zhen Luo, Jing Li, Li Wang

**Affiliations:** 1 Department of Neurology, The First Hospital of Tsinghua University, School of Clinical Medicine, Tsinghua Medicine, Tsinghua University, Beijing, China; 2 Nuclear Medicine Department, The First Hospital of Tsinghua University, School of Clinical Medicine, Tsinghua Medicine, Tsinghua University, Beijing, China; 3 Clinical Laboratory, The First Hospital of Tsinghua University, School of Clinical Medicine, Tsinghua Medicine, Tsinghua University, Beijing, China; 4 Imaging Department, The First Hospital of Tsinghua University, School of Clinical Medicine, Tsinghua Medicine, Tsinghua University, Beijing, China

**Keywords:** basal ganglia calcification, dystonia, homocysteine, PANK2, pantothenate kinase-associated neurodegeneration

## Abstract

**Background:**

Pantothenate kinase-associated neurodegeneration (PKAN) is the most common subtype of neurodegeneration with brain iron accumulation and is classically associated with pallidal iron deposition and the “eye-of-the-tiger” sign on MRI. However, atypical clinicoradiological presentations may complicate recognition.

**Case Presentation:**

We report a 35-year-old woman with adolescent-onset, slowly progressive dystonia characterized by involuntary mouth opening, tongue protrusion, abnormal limb posturing, gait impairment, and cognitive decline. Neuroimaging showed pallidal signal abnormalities compatible with an eye-of-the-tiger-like pattern together with symmetric basal ganglia calcification. Genetic testing identified a homozygous PANK2 variant, NM_001386393.1:c.940C>T [p.(Leu314Phe)], with family segregation consistent with autosomal recessive inheritance. According to the clinical laboratory report, the variant was classified as likely pathogenic under the ACMG/AMP framework (PM2_supporting, PM3_strong, and PP3_moderate). Conservation assessment, computational prediction, and structural modeling supported a deleterious effect, although direct functional validation was not available. Additional findings included acanthocytosis, mild hyperhomocysteinemia, and electrophysiological evidence of peripheral nerve involvement.

**Conclusion:**

This case highlights an atypical but clinically informative PKAN presentation in which pallidal iron deposition coexisted with symmetric basal ganglia calcification. PKAN should remain in the differential diagnosis of slowly progressive dystonia even when neuroimaging is not fully classic. The possible links among CoA dysregulation, iron deposition, calcification, and associated peripheral findings should be regarded as hypothesis-generating.

## Introduction

Dystonia comprises a group of movement disorders characterized by sustained or intermittent muscle contractions that cause abnormal movements and/or postures, and its pathophysiology is closely linked to dysfunction within the basal ganglia–cerebellar–thalamo–cortical network (Yamada, 2021). Among inherited dystonias, pantothenate kinase–associated neurodegeneration (PKAN) is the most common subtype within the spectrum of neurodegeneration with brain iron accumulation (NBIA). PKAN is caused by pathogenic variants in *PANK2* that disrupt coenzyme A (CoA) metabolism, leading to iron deposition in basal ganglia structures (particularly the globus pallidus), neuronal dysfunction, and progressive movement impairment (Sadnicka et al., 2022). Classic PKAN typically presents in childhood or adolescence; on T2-weighted MRI, the characteristic “eye-of-the-tiger” sign is often observed, and patients may also develop dysarthria, pyramidal signs, and cognitive involvement (Choayb et al., 2021).

However, PKAN exhibits marked clinical heterogeneity. In a subset of patients, disease progression is relatively slow and neuroimaging findings may not fully conform to the classic pattern of iron deposition; moreover, so-called “non-core” features—such as basal ganglia calcification, peripheral nervous system involvement, or metabolic abnormalities—may obscure key diagnostic clues and lead to prolonged misdiagnosis or missed diagnosis. We herein report a woman with adolescent-onset disease and a >20-year course whose neuroimaging showed both an eye-of-the-tiger pattern and symmetric bilateral basal ganglia calcification, accompanied by evidence of peripheral nervous system involvement and hyperhomocysteinemia. Genetic testing confirmed a homozygous *PANK2* variant, *PANK2* c.940C>T [(p.Leu314Phe)] (Santambrogio et al., 2020; Cordaro et al., 2021).

The present case is clinically noteworthy because it combines a PKAN-compatible phenotype with an unusual imaging pattern in which pallidal iron deposition coexists with symmetric basal ganglia calcification, a finding that may broaden the differential diagnosis and delay recognition of PKAN. The main purpose of this report is therefore not to establish a definitive molecular mechanism, but to highlight a diagnostically informative atypical presentation and to emphasize that *PANK2*-related disease should still be considered in slowly progressive dystonia even when neuroimaging is not fully classic. Second, the case demonstrates a differential response to symptomatic therapy (Thakur et al., 2021).

## Case Presentation

### Patient information

A 35-year-old woman was admitted for “recurrent involuntary mouth opening with tongue protrusion and progressive gait impairment for more than 20 years” Symptom onset occurred at approximately 16 years of age, initially presenting as intermittent involuntary mouth opening and tongue protrusion accompanied by abnormal gait/posturing and occasional falls. She denied limb numbness or weakness, resting or action tremor, episodic impairment of consciousness, hallucinations, or prominent visual disturbance. She sought medical attention at outside institutions early in the disease course but did not receive a definitive diagnosis; acupuncture and rehabilitation therapies were ineffective. Her symptoms progressed slowly over time. Over the past 5 years, her chewing ability and independent ambulation declined markedly; she was limited to a semi-liquid diet and required assistance from another person or support from surrounding objects to ambulate slowly (Supplementary Videos S1,S2). Approximately 3 years prior to admission, she underwent spinal nerve electrical stimulation at an outside institution without clear symptomatic improvement. She reported frequent falls during the disease course and gradually developed finger joint deformities and abnormal posturing. Secondary amenorrhea developed at 33 years of age. Her parents and brother were reportedly healthy; she denied consanguinity and a family history of similar neurologic disease. To provide a clearer overview of the prolonged disease course, major investigations, and treatment milestones, a clinical timeline is summarized in Table 1.

**TABLE 1:** Clinical timeline of disease progression, investigations, and treatment.

Time point	Clinical events	Clinical significance
Age 16 years	Intermittent involuntary mouth opening, tongue protrusion, abnormal gait/posturing, occasional falls	Adolescent-onset dystonia-like presentation
Early disease course	Local evaluations without definite diagnosis; acupuncture/rehabilitation ineffective	Early diagnostic delay
Progressive course	Gradual worsening of gait and oromandibular dysfunction; frequent falls; finger deformities	Slowly progressive atypical PKAN phenotype
∼5 years before admission	Severe decline in chewing and independent ambulation	Marked functional deterioration
∼3 years before admission	Spinal nerve electrical stimulation without obvious benefit	Limited response to prior symptomatic intervention
1 day before admission	Evaluation at Xuanwu Hospital; diagnosed with ataxia/dystonia; WES initiated	Trigger for etiologic work-up
Current admission	Multimodal diagnostic evaluation completed	Recognition of atypical PKAN constellation
During hospitalization	Multidisciplinary symptomatic treatment	Partial symptomatic benefit
1-month follow-up	Improvement in focal dystonic manifestations, but chewing remained impaired	Persistent disability despite treatment

### Clinical findings

**FIGURE 1:**
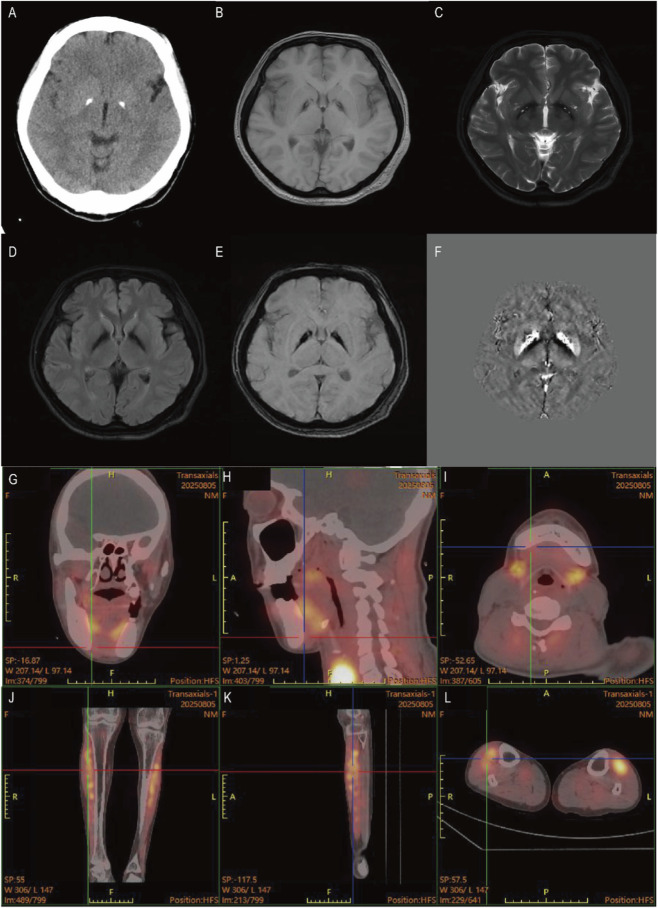
Neuroimaging findings: symmetric basal ganglia calcification and pallidal iron deposition with an “eye-of-the-tiger–like” pattern. **(A)** Non-contrast head CT shows symmetric calcification of the bilateral basal ganglia. **(B)** T1-weighted MRI demonstrates predominantly hypointense signal in the bilateral globus pallidus with a central relative hyperintense focus. **(C)** T2-weighted MRI shows hypointensity of the bilateral globus pallidus with a central punctate relative hyperintensity, consistent with an “eye-of-the-tiger–like” appearance (the central hyperintensity is atypical compared with the classic eye-of-the-tiger-like pattern). **(D)** FLAIR sequence (T2 with CSF suppression) demonstrates hypointensity in the bilateral globus pallidus with a subtle/suspected central relative hyperintensity. **(E)** SWI reveals marked hypointensity of the bilateral globus pallidus, indicating deposition of susceptibility material (suggestive of iron). **(F)** The SWI phase map shows phase changes in the globus pallidus consistent with iron deposition. **(G,H,I)** SPECT/CT demonstrated asymmetric increased radiotracer uptake in the bilateral genioglossus muscles, more pronounced on the left. Mildly increased uptake was also noted in the muscles near the mandibular angle and in the digastric muscles. No appreciable increase in uptake was observed in either the medial or lateral pterygoid muscles bilaterally. **(J,K,L)** SPECT/CT demonstrated increased radiotracer uptake in the bilateral anterior tibial muscle group (tibialis anterior).

**FIGURE 2:**
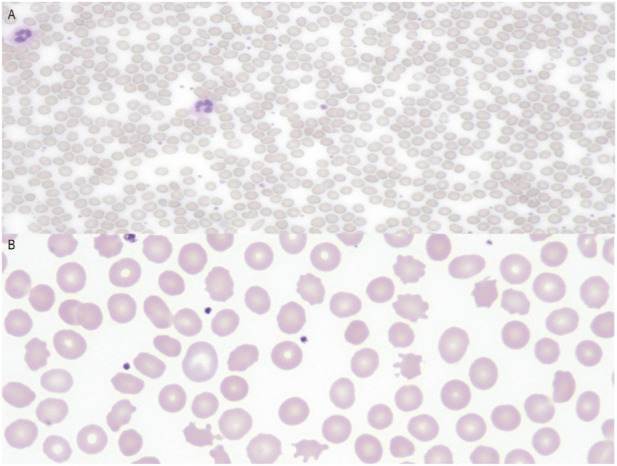
Peripheral blood smear showing acanthocytosis. **(A)** Low-power view demonstrates prominent red blood cell morphological abnormalities with an increased proportion of acanthocytes. **(B)** High-power view shows numerous acanthocytes characterized by irregularly distributed, coarse spicules of variable length on the erythrocyte surface, consistent with acanthocytosis.

On admission, her blood pressure was 142/72 mmHg. Dysarthria and involuntary mouth opening with tongue protrusion were noted. Neurologic examination revealed incomplete eyelid closure bilaterally, distal weakness of both upper limbs (finger abduction/adduction strength approximately Medical Research Council grade 3), increased muscle tone in the right limbs, and deformities of both hands with striatal hand/foot–like posturing. Deep tendon reflexes were decreased in the upper limbs and brisk in the lower limbs. Coordination testing was limited because she was unable to stand independently for full assessment. Non-contrast head CT demonstrated symmetric calcification of the bilateral basal ganglia. On MRI, T2/FLAIR sequences showed diffuse hypointensity of the globus pallidus with a central punctate relative hyperintensity; together with marked hypointensity on susceptibility-weighted imaging (SWI), these findings were consistent with an eye-of-the-tiger-like pattern in the context of NBIA-spectrum iron deposition, although the central hyperintensity was atypical and accompanied by basal ganglia calcification, suggesting an atypical imaging constellation (Figures 1A–F). SPECT/CT demonstrated increased radiotracer uptake in selected muscle groups. Uptake was elevated in portions of the suprahyoid muscles (genioglossus, mylohyoid, and digastric), most prominently in the left genioglossus, whereas no appreciable increase was observed in the bilateral medial or lateral pterygoid muscles. Additional increased uptake was noted in the bilateral supraspinatus and splenius muscles, the right popliteus, and the bilateral anterior tibial muscle group (tibialis anterior), consistent with hyperactivity of clinically involved muscles(Figures 1G–L). Spine radiographs showed degenerative changes and postoperative findings. Neuropsychological assessment demonstrated multidomain cognitive impairment (MoCA 15; MMSE 19), involving immediate and delayed recall, repetition, abstraction, visuospatial/executive function, calculation, orientation, attention, verbal fluency, and naming. Laboratory testing revealed acanthocytosis on peripheral blood smear (Figure 2) and mildly elevated homocysteine (21.3 μmol/L). Thyroid function, vitamin B12, and serum electrolytes were within normal limits, and the folate level was 8.53 nmol/L. Neuropsychological assessment demonstrated multidomain cognitive impairment. Electrophysiological testing demonstrated involvement of the left ulnar motor nerve and bilateral ulnar sensory nerves, providing objective evidence of peripheral nerve involvement.

### Genetic testing

**FIGURE 3:**
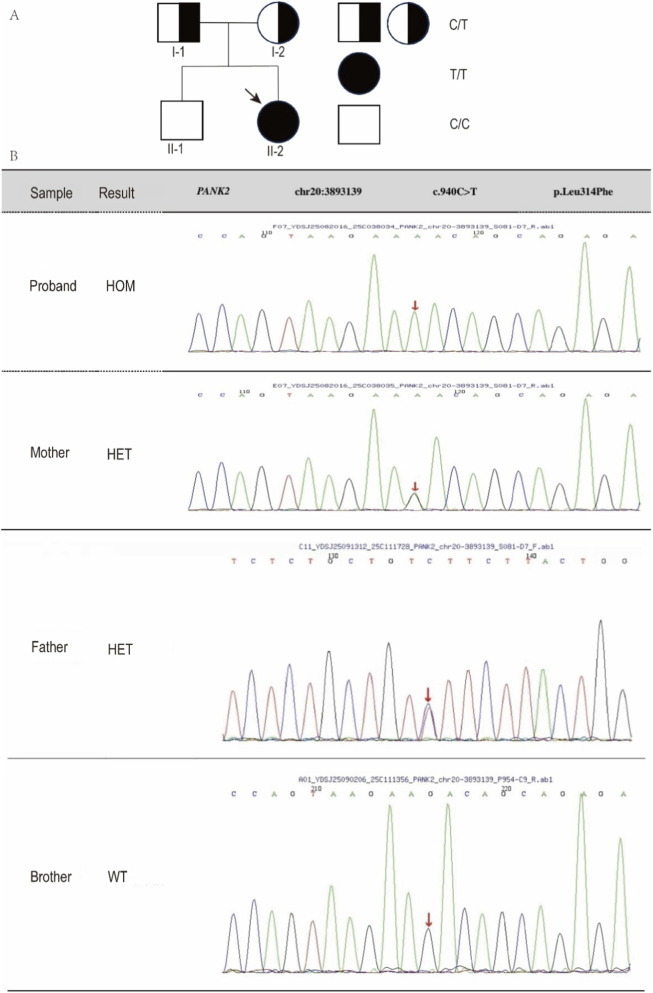
**(A)** Pedigree chart. Pedigree showing segregation of the *PANK2* NM_001386393.1:c.940C>T [p.(Leu314Phe)] variant in the family. The proband is homozygous (T/T), both parents are heterozygous carriers (C/T), and the unaffected brother is wild type (C/C), consistent with autosomal recessive inheritance. **(B)** Family segregation of the *PANK2* variant (Sanger sequencing chromatograms). Sanger sequencing chromatograms of the *PANK2* c.940C>T (p.Leu314Phe) locus are shown for the proband and first-degree relatives. The proband carries the variant, whereas both parents are heterozygous carriers (C/T), supporting an autosomal-recessive inheritance pattern. The proband’s brother is wild type (C/C) and does not carry the variant.

Genetic testing identified a homozygous(HOM) *PANK2* variant, NM_001386393.1:c.940C>T [p.(Leu314Phe)]. Family segregation analysis showed that both parents were heterozygous(HET) carriers and that the patient’s brother was wild type(WT), supporting autosomal recessive inheritance(Figure 3). According to the clinical laboratory report, this variant was classified as likely pathogenic under the ACMG framework (PM2_supporting, PM3_strong, and PP3_moderate). Population database review, conservation analysis, and computational prediction supported a deleterious effect. Structural modeling further suggested that the p.(Leu314Phe) substitution may reduce local protein stability (Table 2). However, no direct functional assay was performed, and the biological consequences of this variant remain inferential rather than experimentally established.

**TABLE 2:** Structured interpretation of the *PANK2* NM_001386393.1:c.940C>T [p.(Leu314Phe)] variant.

Item	Result
Gene	*PANK2*
Transcript	NM_001386393.1
Nucleotide change	c.940C>T
Protein change	p.(Leu314Phe)
Variant type	Missense
Zygosity	Homozygous
Inheritance pattern	Autosomal recessive
Segregation	Proband homozygous; both parents heterozygous; brother wild type
Population frequency	1000 genomes project, ESP6500, ExAC, and ExAC-EAS, and the variant was recorded as not observed
Conservation	Conserved residue/site
In silico prediction	Revel: Deleterious; PolyPhen-2: Potentially damaging effect
Structural modeling	Predicted reduction in local protein stability; ΔΔG = −0.6 kcal/mol(Dynamut2)
ACMG evidence	PM2_supporting, PM3_strong, PP3_moderate
Final classification	Likely pathogenic

At the protein level, the substitution replaces leucine with a bulkier aromatic phenylalanine residue, which may alter local packing and stability; however, this inference is computational and should be interpreted cautiously.

Conservation analysis indicated that the c.940C>T site is highly conserved (Supplementary Table S1). Protein stability modeling yielded a ΔΔG (mutant − wild type) of −0.6 kcal/mol, suggesting reduced local structural stability. At the molecular level, p. Leu314Phe represents a “hydrophobic-to-hydrophobic” substitution; however, the two residues differ substantially in side-chain chemistry. Leucine carries a branched aliphatic hydrocarbon side chain (–CH_2_–CH(CH_3_)_2_) with greater conformational flexibility, whereas phenylalanine contains an aromatic phenyl ring (–CH_2_–C_6_H_5_) that is bulkier and more rigid and may introduce π-related interactions. Accordingly, this substitution could increase local steric constraints, alter the packing of the native hydrophobic core, and/or perturb neighboring side-chain orientations and secondary-structure stability, thereby reducing local conformational stability (consistent with the stability prediction) and ultimately compromising *PANK2* folding efficiency and/or enzymatic activity.

### Therapeutic interventions

During hospitalization, the patient received multidisciplinary management: (1) Symptomatic antispastic therapy with baclofen 5 mg three times daily and clonazepam 0.25 mg at bedtime (with planned dose titration and monitoring for adverse effects). (2) Nutritional and swallowing management, including an individualized enteral nutrition regimen and assessment of swallowing function and aspiration risk. (3) Botulinum toxin therapy: Target muscles and salivary glands were localized under combined electromyography (EMG) guidance and color Doppler ultrasonography, followed by botulinum toxin type A injection. To address oromandibular dystonia and related functional limitation, multipoint injections were performed in the bilateral lateral pterygoid muscles, genioglossus, digastric, platysma, and mentalis. Given excessive sialorrhea/salivary hypersecretion, intraglandular injections were administered into the bilateral parotid and submandibular glands. To alleviate abnormal lower-limb posturing/spasticity, additional injections were delivered to the extensor hallucis longus, extensor digitorum longus, and tibialis anterior. The total dose of botulinum toxin type A was 200 units, administered across multiple sites. (4) Metabolic intervention for hyperhomocysteinemia with methylcobalamin, vitamin B6, and folic acid supplementation, with a follow-up plan for repeat laboratory testing.

## Follow-up and outcomes

After discharge, continued antispastic therapy was recommended with gradual dose adjustment based on efficacy and tolerability, along with ongoing rehabilitation and fall-prevention measures. Serial monitoring of homocysteine and relevant vitamin levels was advised, together with reassessment of nutritional status. Supportive management for peripheral nervous system involvement—including neurotrophic therapy and treatment of pain or sensory symptoms—was recommended as indicated. From a genetic standpoint, family members were advised to undergo genetic counseling and reproductive risk assessment. At 1-month follow-up, the patient showed improvement in lower-limb striatal toe posturing and oromandibular involuntary movements, with reduced sialorrhea; however, improvement in chewing remained limited, and enteral feeding with intensified nutritional support was still required. Although local botulinum toxin injection alleviated some focal dystonic manifestations, its overall benefit remained limited in the context of generalized dystonia.

## Discussion

PKAN is the most common subtype within the NBIA spectrum and is classically characterized by progressive dystonia, pyramidal signs, cognitive impairment, and pallidal iron deposition with the “eye-of-the-tiger” sign on MRI (Chang et al., 2020; Jeong et al., 2019). However, substantial clinical heterogeneity has been recognized, particularly in atypical or later-onset cases, in which disease progression may be relatively slow and early manifestations may be nonspecific (Chang et al., 2020; Kastaniotis et al., 2021). In the present patient, symptom onset occurred in adolescence and the disease course extended over more than 20 years, with initial manifestations dominated by oromandibular dystonia and gait abnormality. This pattern is clinically important because it can easily be misclassified as primary dystonia in the early stage, thereby delaying recognition of PKAN (Kastaniotis et al., 2021; Jain et al., 2021). The major educational value of this report, therefore, lies not in proposing a definitive new mechanism, but in highlighting a diagnostically informative atypical presentation of PKAN.

The most striking feature of this case is the coexistence of pallidal iron deposition and symmetric basal ganglia calcification. In clinical practice, basal ganglia calcification often redirects the differential diagnosis toward disorders of calcium-phosphate metabolism, infectious or toxic etiologies, or primary familial brain calcification. Symmetric basal ganglia calcification in this case prompted careful consideration of alternative etiologies. Infectious causes were not supported by the absence of relevant exposure history and by unremarkable inflammatory/immune screening. Toxic exposure was not supported by the personal history. Common systemic metabolic or endocrine explanations were not supported by the available biochemical evaluation, including normal electrolytes and thyroid-related testing. Mitochondrial disease was considered less likely because mitochondrial genome analysis was negative. Nevertheless, calcification has been reported in PKAN, although it is not a core radiological feature (Fasano et al., 2017). In this context, our case reinforces an important practical point: PKAN should remain in the differential diagnosis of adolescent- or young adult-onset slowly progressive dystonia even when CT demonstrates basal ganglia calcification and MRI is not fully classic. At the same time, the significance of calcification in this setting should be interpreted cautiously. Although previous work has suggested a potentially harmful iron-calcium relationship in PKAN and raised the possibility that mitochondrial stress, oxidative injury, and dysregulated mineral homeostasis may contribute to secondary mineralization (Santambrogio et al., 2020), the present case does not allow direct mechanistic inference. Accordingly, the proposed relationship between iron deposition and calcification in this patient should be regarded as hypothesis-generating rather than established.

The genetic finding in this case provides important diagnostic support. The patient harbored a homozygous *PANK2* c.940C>T [p.(Leu314Phe)] variant, and segregation analysis was consistent with autosomal recessive inheritance. According to the clinical laboratory report, this variant was classified as likely pathogenic under the ACMG/AMP framework based on PM2_supporting, PM3_strong, and PP3_moderate. In addition, the affected residue is conserved, computational prediction supports a deleterious effect, and structural modeling suggests reduced local protein stability. These findings are biologically plausible in light of previous work showing that CoA deficiency in *PANK2*-related disease is linked to mitochondrial dysfunction, oxidative stress, and abnormal iron handling (Jeong et al., 2019; Orellana et al., 2016; Hayflick et al., 2022). However, the current evidence remains indirect. No enzyme activity assay or other functional validation was performed, and we therefore avoided interpreting p.(Leu314Phe) as having a definitively established pathogenic mechanism. The value of the genetic result in this report is primarily diagnostic and supportive rather than mechanistically confirmatory.

Several associated findings in this patient also merit discussion, but they should be interpreted cautiously. First, acanthocytosis was documented on peripheral blood smear. This is compatible with prior literature indicating that erythrocyte membrane abnormalities may occur in *PANK2*-related disease and may reflect broader disturbances in lipid metabolism and membrane homeostasis (Werning et al., 2022). Second, mild hyperhomocysteinemia was observed. Because thyroid function, vitamin B12, and serum electrolytes were within normal limits, and the folate level was 8.53 nmol/L, some common secondary explanations appear less likely; however, the cause and clinical significance of this finding could not be fully established. Third, unlike the more speculative wording in the previous version, peripheral involvement in this patient was supported by electrophysiological abnormalities involving the left ulnar motor nerve and bilateral ulnar sensory nerves. However, this finding should be interpreted conservatively and should not be taken to indicate that peripheral neuropathy is an established component of PKAN. Large case series and reviews of PKAN have emphasized dystonia, gait impairment, pyramidal signs, and cognitive or psychiatric features, but have not identified peripheral neuropathy or calcification as central manifestations (Fasano et al., 2017; Shalash et al., 2021; Pan and Zhu, 2020). Accordingly, rather than incorporating these findings into a broader multisystem pathogenic model, we present them as associated observations of possible clinical relevance that may increase awareness of phenotypic variability in PKAN.

From a management perspective, this case also illustrates the limitations of symptomatic therapy in atypical PKAN. Botulinum toxin injections improved lower-limb dystonic posturing to some extent, but chewing dysfunction remained severe. This pattern is consistent with the broader therapeutic challenge in PKAN, where symptomatic benefit is often partial and longitudinal multidisciplinary care is required (Chan et al., 2024). Mechanism-based therapeutic strategies targeting the CoA biosynthetic defect, including pantothenate-related or mitochondrial approaches, remain of considerable interest (Thakur et al., 2021; Hayflick et al., 2022). However, such strategies are still investigational, and this single case cannot support variant-specific therapeutic extrapolation. More broadly, genotype-phenotype correlation remains clinically useful not because it enables immediate precision therapy in most cases, but because it may improve diagnostic confidence, refine counseling, and help frame future studies of targeted intervention (Li et al., 2022).

This report has several limitations. First, the biological effect of p.(Leu314Phe) was inferred from segregation and computational evidence rather than direct functional testing. Second, although the coexistence of calcification and pallidal iron deposition is clinically striking, a complete dedicated exclusion work-up for all alternative causes of basal ganglia calcification was not fully available. Third, although peripheral nerve involvement was electrophysiologically supported, its mechanistic relationship to *PANK2*-associated disease remains uncertain. Despite these limitations, this case contributes a clinically useful reminder that PKAN may present with an atypical clinicoradiological constellation, and that integrated assessment of phenotype, neuroimaging, electrophysiology, and molecular genetics is essential in complex movement-disorder presentations.

## Data Availability

The original contributions presented in the study are included in the article/Supplementary Material, further inquiries can be directed to the corresponding author.
