# Supplementary material for: Case Report: clinical manifestations and imaging features associated with PANK2 c.940C>T variant in PKAN with symmetric basal ganglia calcification
Source: Front Genet. 2026 Jun 10;17:1792232. doi: 10.3389/fgene.2026.1792232 (PMC13290116; doi:10.3389/fgene.2026.1792232)
Supplement: Supplementary file 1 [file Table1.docx]

| [QUERY](http://www.uniprot.org/uniprot/Q9BZ23) | SILAVYSKDNY--------KRVTGTSLGGGTFFGLCC | L | LTGCTTFEEALEMASRGDSTKVDKLVRDIYG-G---DY |
| --- | --- | --- | --- |
| [sp\|G1TMH5#1](http://www.uniprot.org/uniprot/G1TMH5) | SILAVYSKDNY--------KRVTGTSLGGGTFFGLCC | L | LTGCTTFEEALEMASRGDSTKVDKLVRDIYG-G---DY |
| [sp\|Q7M753#1](http://www.uniprot.org/uniprot/Q7M753) | SILAVYSKDNY--------KRVTGTSLGGGTFFGLCC | L | LTGCSTFEEALEMASRGDSTKVDKLVRDIYG-G---DY |
| [sp\|F6S4K6#1](http://www.uniprot.org/uniprot/F6S4K6) | SILAVYSKDNY--------KRVTGTSLGGGTFFGLCC | L | LTGGLHSEEALEMASRGDSTKVDKLVRDIYG-G---DY |
| [sp\|UPI000194C70D#1](http://www.uniprot.org/uniparc/UPI000194C70D) | SILAVYSKDNY--------KRVTGTSLGGGTFFGLCC | L | LTGCSTFEEALEMASHGDSTKVDKLVRDIYG-G---DY |
| [sp\|Q3TIG7#1](http://www.uniprot.org/uniprot/Q3TIG7) | SILAVYSKDNY--------KRVTGTSLGGGTFFGLCC | L | LTGCSTFEEALEMASRGDSTKVDKLVRDIYG-G---DY |
| [sp\|G1NKK3#1](http://www.uniprot.org/uniprot/G1NKK3) | SILAVYSKENY--------RWVTGTSLGGGTFFGLCC | L | LTGCSTFEEAMEMASHGDSTKVDKLVRDIYG-G---DY |
| [sp\|UPI0002035339#1](http://www.uniprot.org/uniparc/UPI0002035339) | SILAVYSKENY--------RWVTGTSLGGGTFFGLCC | L | LTGCSTFEEAMEMASHGDSTKVDKLVRDIYG-G---DY |
| [sp\|UPI00022B00B6#1](http://www.uniprot.org/uniparc/UPI00022B00B6) | SILAVYSEDNY--------KRVTGTSLGGGTFLGLCC | L | LTGCSTFEEALEMASQGESTRVDKLVRDIYG-G---DY |
| [sp\|F6XZX1#1](http://www.uniprot.org/uniprot/F6XZX1) | SILAVHSKDNY--------KRVTGTSLGGGTFLGLCS | L | LTGCGSFEEALEMASKGDSTQADKLVRDIYG-G---DY |
| [sp\|F7DCT5#1](http://www.uniprot.org/uniprot/F7DCT5) | SILAVYSKDNY--------KRVTGTSLGGGTFLGLCC | L | LTGCETFEEALEMAAKGDSTNVDKLVKDIYG-G---DY |
| [sp\|F6X0R4#1](http://www.uniprot.org/uniprot/F6X0R4) | SILAVYSKDNY--------KRVTGTSLGGGTFFGLCC | L | LTGCSTFEEALEMASLGDSTKVDKLVRDIYG-G---DY |
| [sp\|UPI00020F5CD6#1](http://www.uniprot.org/uniparc/UPI00020F5CD6) | SILAVYSKDNY--------KRVTGTSLGGGTFLGLCC | L | LTGCETFEEALEMAAKGDSTNVDKLVKDIYG-G---DY |
| [sp\|F7FBT3#1](http://www.uniprot.org/uniprot/F7FBT3) | SILAVYSKDNY--------KRVTGTSLGGGTFLGLCC | L | LTGCETFEEALEMAAKGDSTNVDKLVKDIYG-G---DY |
| [sp\|G1T3F1#1](http://www.uniprot.org/uniprot/G1T3F1) | SILAVYSKDNY--------KRVTGTSLGGGTFLGLCC | L | LTGCETFEEALEMAAKGDSTNVDKLVKDIYG-G---DY |
| [sp\|UPI0001E88770#1](http://www.uniprot.org/uniparc/UPI0001E88770) | SILAVYSKDNY--------KRVTGTSLGGGTFLGLCC | L | LTGCETFEEALEMAAKGDSTNVDKLVKDIYG-G---DY |
| [sp\|D3ZIP5#1](http://www.uniprot.org/uniprot/D3ZIP5) | SILAVYSKDNY--------KRVTGTSLGGGTFLGLCC | L | LTGCETFEEALDMAAKGDSTNVDKLVKDIYG-G---DY |
| [sp\|Q8K4K6#1](http://www.uniprot.org/uniprot/Q8K4K6) | SILAVYSKDNY--------KRVTGTSLGGGTFLGLCC | L | LTGCETFEEALDMAAKGDSTNVDKLVKDIYG-G---DY |
| [sp\|E1BCR9#1](http://www.uniprot.org/uniprot/E1BCR9) | SILAVYSKDNY--------KRVTGTSLGGGTFLGLCC | L | LTGCETFEEALEMAAKGDSTNVDKLVKDIYG-G---DY |
| [sp\|UPI0001D56C61#1](http://www.uniprot.org/uniparc/UPI0001D56C61) | SILAVYSKDNY--------KRVTGTSLGGGTFLGLCC | L | LTGCETFEEALEMAAKGDSTNVDKLVKDIYG-G---DY |
| [sp\|G1KL97#1](http://www.uniprot.org/uniprot/G1KL97) | SILAVHSKDNY--------KRVTGTSLGGGTFLGLCS | L | LTGCESFEEALEMASKGDSTHADKLVRDIYG-G---DY |
| [sp\|G1NY47#1](http://www.uniprot.org/uniprot/G1NY47) | SILAVYSKDNY--------KRVTGTSLGGGTFLGLCC | L | LTGCETFEEAMEMAAKGDSTNVDKLVKDIYG-G---DY |
| [sp\|UPI000223411A#1](http://www.uniprot.org/uniparc/UPI000223411A) | SILAVYSKDNY--------KRVTGTSLGGGTFLGLCC | L | LTGCETFEEALEMAAKGDSTNVDKLVKDIYG-G---DY |
| [sp\|UPI000036E83B#1](http://www.uniprot.org/uniparc/UPI000036E83B) | SILAVYSKDNY--------KRVTGTSLGGGTFLGLCC | L | LTGCETFEEALEMAAKGDSTNVDKLVKDIYG-G---DY |
| [sp\|Q8TE04#1](http://www.uniprot.org/uniprot/Q8TE04) | SILAVYSKDNY--------KRVTGTSLGGGTFLGLCC | L | LTGCETFEEALEMAAKGDSTNVDKLVKDIYG-G---DY |
| [sp\|G1RNZ2#1](http://www.uniprot.org/uniprot/G1RNZ2) | SILAVYSKDNY--------KRVTGTSLGGGTFLGLCC | L | LTGCETFEEALEMAAKGDSTNVDKLVKDIYG-G---DY |
| [sp\|G3QFR3#1](http://www.uniprot.org/uniprot/G3QFR3) | SILAVYSKDNY--------KRVTGTSLGGGTFLGLCC | L | LTGCETFEEALEMAAKGDSTNVDKLVKDIYG-G---DY |
| [sp\|F7ERF8#1](http://www.uniprot.org/uniprot/F7ERF8) | SILAVYSKDNY--------KRVTGTSLGGGTFLGLCC | L | LTGCETFEEALEMAAKGDSTNVDKLVKDIYG-G---DY |
| [sp\|F7GUZ3#1](http://www.uniprot.org/uniprot/F7GUZ3) | SILAVYSKDNY--------KRVTGTSLGGGTFLGLCC | L | LTGCETFEEALEMAAKGDSTNVDKLVKDIYG-G---DY |
| [sp\|F7F4G9#1](http://www.uniprot.org/uniprot/F7F4G9) | SILAVYSKDNY--------KRVTGTSLGGGTFLGLCC | L | LTGCETFEEALEMAAKGDSTNVDKLVKDIYG-G---DY |
| [sp\|B3KWN3#1](http://www.uniprot.org/uniprot/B3KWN3) | SILAVYSKDNY--------KRVTGTSLGGGTFLGLCC | L | LTGCETFEEALEMAAKGDSTNVDKLVKDIYG-G---DY |
| [sp\|G3RXB0#1](http://www.uniprot.org/uniprot/G3RXB0) | SILAVYSKDNY--------KRVTGTSLGGGTFLGLCC | L | LTGCETFEEALEMAAKGDSTNVDKLVKDIYG-G---DY |
| [sp\|UPI0001DEADE3#1](http://www.uniprot.org/uniparc/UPI0001DEADE3) | SILAVHSKDNY--------KRVTGTSLGGGTFLGLCC | L | LTGCESFEEALEMASKGDSTQADKLVRDIYG-G---DY |
| [sp\|E2QVM2#1](http://www.uniprot.org/uniprot/E2QVM2) | SILAVHSKDNY--------KRVTGTSLGGGTFLGLCC | L | LTGCESFEEALEMASKGDSTQADKLVRDIYG-G---DY |
| [sp\|UPI0001DEA9BA#1](http://www.uniprot.org/uniparc/UPI0001DEA9BA) | SILAVYSKDNY--------KRVTGTSLGGGTFLGLCC | L | LTGCETFEEALEMAAKGDSTNVDKLVKDIYG-G---DY |
| [sp\|UPI000184EC3C#1](http://www.uniprot.org/uniparc/UPI000184EC3C) | SILAVYSKDNY--------KRVTGTSLGGGTFLGLCC | L | LTGCETFEEALEMAAKGDSTNVDKLVKDIYG-G---DY |
| [sp\|F1QEN2#1](http://www.uniprot.org/uniprot/F1QEN2) | SILAVYSKDDY--------KRVTGTSLGGGTFLGLCC | L | LTGCETFEEALEMAAKGDSTNVDKLVKDIYG-G---DY |
| [sp\|A2RV28#1](http://www.uniprot.org/uniprot/A2RV28) | SILAVYSKDDY--------KRVTGTSLGGGTFLGLCC | L | LTGCETFEEALEMAAKGDSTNVDKLVKDIYG-G---DY |
| [sp\|G1LKN9#1](http://www.uniprot.org/uniprot/G1LKN9) | SILAVHSKDNY--------KRVTGTSLGGGTFLGLCC | L | LTGCESFEEALEMASKGDSTQADKLVRDIYG-G---DY |
| [sp\|Q08DA5#1](http://www.uniprot.org/uniprot/Q08DA5) | SILAVHSKDNY--------KRVTGTSLGGGTFLGLCS | L | LTGCESFEEALEMASKGDSTQADKLVRDIYG-G---DY |
| [sp\|E1BWU5#1](http://www.uniprot.org/uniprot/E1BWU5) | SILSVHSKDNY--------KRVTGTSLGGGTFLGLCS | L | LTGCESFEEALEMASKGDSTHADKLVRDIYG-G---DY |
| [sp\|UPI00022F4113#1](http://www.uniprot.org/uniparc/UPI00022F4113) | SILAVYSKDNY--------KRVTGTSLGGGTFLGLCC | L | LTGCETFEEALEMAAKGDSTNVDKLVKDIYG-G---DY |
| [sp\|UPI0002036409#1](http://www.uniprot.org/uniparc/UPI0002036409) | SILSVHSKDNY--------KRVTGTSLGGGTFLGLCS | L | LTGCESFEEALEMASKGDSTHADKLVRDIYG-G---DY |
| [sp\|G3WUD1#1](http://www.uniprot.org/uniprot/G3WUD1) | SILAVHSKDNY--------KRVTGTSLGGGTFLGLCS | L | LTGCESFEEALEMASKGESTYADKLVRDIYG-G---DY |
| [sp\|G5BP50#1](http://www.uniprot.org/uniprot/G5BP50) | SILAVHSKDNY--------KRVTGTSLGGGTFLGLCS | L | LTGCESFEEALEMASKGDSTQADKLVRDIYG-G---DY |
| [sp\|UPI00022B5096#1](http://www.uniprot.org/uniparc/UPI00022B5096) | SILAVHSKDNY--------KRVTGTSLGGGTFLGLCS | L | LTGCESFEEALEMASKGDSTQADKLVRDIYG-G---DY |
| [sp\|F1RR90#1](http://www.uniprot.org/uniprot/F1RR90) | SILAVHSKDNY--------KRVTGTSLGGGTFLGLCS | L | LTGCESFEEALEMASKGDSTQADKLVRDIYG-G---DY |
| [sp\|UPI0001DE7E38#1](http://www.uniprot.org/uniparc/UPI0001DE7E38) | SILAVYSKDNY--------KRVTGTSLGGGTFLGLCC | L | LTGCETFEEALEMAAKGDSTNVDKLVKDIYG-G---DY |
| [sp\|F6XS39#1](http://www.uniprot.org/uniprot/F6XS39) | SILAVHSKDNY--------KRVTGTSLGGGTFLGLCS | L | LTGCESFEEALEMASKGDSTHADKLVRDIYG-G---DY |
| [sp\|F1NL18#1](http://www.uniprot.org/uniprot/F1NL18) | SILSVHSKDNY--------KRVTGTSLGGGTFLGLCS | L | LTGCESFEEALEMASKGDSTHADKLVRDIYG-G---DY |
| [sp\|G1MU06#1](http://www.uniprot.org/uniprot/G1MU06) | SILSVHSKDNY--------KRVTGTSLGGGTFLGLCS | L | LTGCESFEEALEMASKGDSTHADKLVRDIYG-G---DY |
| [sp\|Q9H999#1](http://www.uniprot.org/uniprot/Q9H999) | SILAVHSKDNY--------KRVTGTSLGGGTFLGLCS | L | LTGCESFEEALEMASKGDSTQADKLVRDIYG-G---DY |
| [sp\|G3QQI1#1](http://www.uniprot.org/uniprot/G3QQI1) | SILAVHSKDNY--------KRVTGTSLGGGTFLGLCS | L | LTGCESFEEALEMASKGDSTQADKLVRDIYG-G---DY |
| [sp\|F6VX24#1](http://www.uniprot.org/uniprot/F6VX24) | SILAVHSKDNY--------KRVTGTSLGGGTFLGLCS | L | LTGCESFEEALEMASKGDSTQADKLVRDIYG-G---DY |
| [sp\|F6XZV6#1](http://www.uniprot.org/uniprot/F6XZV6) | SILAVHSKDNY--------KRVTGTSLGGGTFLGLCS | L | LTGCGSFEEALEMASKGDSTQADKLVRDIYG-G---DY |
| [sp\|UPI00016E1300#1](http://www.uniprot.org/uniparc/UPI00016E1300) | SILAVYSENNY--------KRVTGTSLGGGTFLGLCC | L | LTGCSTFDEALQMASQGESTRVDKLVRDIYG-G---DY |
| [sp\|UPI0002035CDF#1](http://www.uniprot.org/uniparc/UPI0002035CDF) | SILAVYSKDNY--------KRVTGSSLGGGTFLGLCC | L | LTGCETFEEALEMAAKGDSTNVDKLVKDIYG-G---DY |
| [sp\|E1BS92#1](http://www.uniprot.org/uniprot/E1BS92) | SILAVYSKDNY--------KRVTGSSLGGGTFLGLCC | L | LTGCETFEEALEMAAKGDSTNVDKLVKDIYG-G---DY |
| [sp\|F7A795#1](http://www.uniprot.org/uniprot/F7A795) | SILAVYSKDNY--------KRVTGTSLGGGTFLGLCC | L | LTGCETFEEALDMAAKGDSTNVDKLVKDIYG-G---DY |
| [sp\|G1TSZ8#1](http://www.uniprot.org/uniprot/G1TSZ8) | SILAVHSKDNY--------KRVTGTSLGGGTFLGLCS | L | LTGCESFEEALEMASKGDSTQADKLVRDIYG-G---DY |
| [sp\|G1NZF1#1](http://www.uniprot.org/uniprot/G1NZF1) | SILAVHSKDNY--------KRVTGTSLGGGTFLGLCS | L | LTGCESFEEALEMASKGDSTHADKLVRDIYG-G---DY |
| [sp\|F1SCX8#1](http://www.uniprot.org/uniprot/F1SCX8) | SILAVYSKDNY--------KRVTGTSLGGGTFLGLCC | L | LTGCETFEEALEMAAKGDSTNVDKLVKDIYG-G---DY |
| [sp\|F7FBU2#1](http://www.uniprot.org/uniprot/F7FBU2) | SILAVYSKDNY--------KRVTGTSLGGGTFLGLCC | L | LTGCETFEEALEMAAKGDSTNVDKLVKDIYG-G---DY |
| [sp\|G3TGT8#1](http://www.uniprot.org/uniprot/G3TGT8) | SVLAVHSKDNY--------KRVTGTSLGGGTFLGLCS | L | LTGCESFEEALEMASKGDSTQADKLVRDIYG-G---DY |
| [sp\|D3ZUQ7#1](http://www.uniprot.org/uniprot/D3ZUQ7) | SILAVHSKDNY--------KRVTGTSLGGGTFLGLCS | L | LTGCESFEEALEMASKGDSTQADRLVRDIYG-G---DY |
| [sp\|UPI000194D1D7#1](http://www.uniprot.org/uniparc/UPI000194D1D7) | SILSVHSKDNY--------KRVTGTSLGGGTFLGLCS | L | LTGCESFEEALEMASKGDSTHADKLVRDIYG-G---DY |
| [sp\|G1N8J8#1](http://www.uniprot.org/uniprot/G1N8J8) | SILAVYSKDNY--------KRVTGSSLGGGTFLGLCC | L | LTGCETFEEALEMAAKGDSTNVDKLVKDIYG-G---DY |
| [sp\|F1PWE4#1](http://www.uniprot.org/uniprot/F1PWE4) | SILAVYSKDNY--------KRVTGTSLGGGTFLGLCC | L | LTGCETFEEALEMAAKGDSTNVDKLVKDIYG-G---DY |
| [sp\|UPI00005A4E35#1](http://www.uniprot.org/uniparc/UPI00005A4E35) | SILAVYSKDNY--------KRVTGTSLGGGTFLGLCC | L | LTGCETFEEALEMAAKGDSTNVDKLVKDIYG-G---DY |
| [sp\|Q7M752#1](http://www.uniprot.org/uniprot/Q7M752) | SILAVHSKDNY--------KRVTGTSLGGGTFLGLCS | L | LTGCESFEEALEMASKGDSTQADRLVRDIYG-G---DY |
| [sp\|Q8R2W9#1](http://www.uniprot.org/uniprot/Q8R2W9) | SILAVHSKDNY--------KRVTGTSLGGGTFLGLCS | L | LTGCESFEEALEMASKGDSTQADRLVRDIYG-G---DY |
| [sp\|UPI000194C818#1](http://www.uniprot.org/uniparc/UPI000194C818) | SILAVYSKDNY--------KRVTGSSLGGGTFLGLCC | L | LTGCETFEEALEMAAKGDSTNVDKLVKDIYG-G---DY |
| [sp\|G3NM99#1](http://www.uniprot.org/uniprot/G3NM99) | SILAVYSETNY--------KRVTGTSLGGGTFLGLCC | L | LTGCSTFEEALEMASQGESTRVDKLVRDIYG-G---DY |
| [sp\|G1SIA8#1](http://www.uniprot.org/uniprot/G1SIA8) | SILAVYSKDNY--------KRVTGTSLGGGTFLGLCC | L | LTGCETFEEALEMAAKGDSTNVDKLVKDIYG-G---DY |
| [sp\|F6XZU5#1](http://www.uniprot.org/uniprot/F6XZU5) | SILAVHSKDNY--------KRVTGTSLGGGTFLGLCS | L | LTGCGSFEEALEMASKGDSTQADKLVRDIYG-G---DY |
| [sp\|UPI000223E3D0#1](http://www.uniprot.org/uniparc/UPI000223E3D0) | SILAVHSKDNY--------KRVTGTSLGGGTFLGLCS | L | LTGCGSFEEALEMASKGDSTQADKLVRDIYG-G---DY |
| [sp\|E7F0R7#1](http://www.uniprot.org/uniprot/E7F0R7) | SILAVNSKDNY--------KRVTGTSLGGGTFLGLCC | L | LTGCETFEEALEMASKGDSTNVDKLVKDIYG-G---DY |
| [sp\|D3ZWD3#1](http://www.uniprot.org/uniprot/D3ZWD3) | SILAVYSKDNY--------KRVTGTSLGGGTFLGLCC | L | LTGCETFEEALDMAAKGDSTNVDKLVKDIYG-G---DY |
| [sp\|Q543J7#1](http://www.uniprot.org/uniprot/Q543J7) | SILAVYSKDNY--------KRVTGTSLGGGTFLGLCC | L | LTGCETFEEALDMAAKGDSTNVDKLVKDIYG-G---DY |
| [sp\|G3TUK3#1](http://www.uniprot.org/uniprot/G3TUK3) | SVLAVHSKDNY--------KRVTGTSLGGGTFLGLCS | L | LTGCESFEEALEMASKGDSTQADKLVRDIYG-G---DY |
| [sp\|Q8TE04-2#1](http://www.uniprot.org/uniprot/Q8TE04) | SILAVYSKDNY--------KRVTGTSLGGGTFLGLCC | L | LTGCETFEEALEMAAKGDSTNVDKLVKDIYG-G---DY |
| [sp\|F7F4W0#1](http://www.uniprot.org/uniprot/F7F4W0) | SILAVYSKDNY--------KRVTGTSLGGGTFLGLCC | L | LTGCETFEEALEMAAKGDSTNVDKLVKDIYG-G---DY |
| [sp\|G3TFR1#1](http://www.uniprot.org/uniprot/G3TFR1) | SILAVYSKDNY--------KRVTGTSLGGGTFLGLCC | L | LTGCETFEEALEMAAKGDSTNVDKLVKDIYG-G---DY |
| [sp\|Q8TE04-4#1](http://www.uniprot.org/uniprot/Q8TE04) | SILAVYSKDNY--------KRVTGTSLGGGTFLGLCC | L | LTGCETFEEALEMAAKGDSTNVDKLVKDIYG-G---DY |
| [sp\|G1MEH7#1](http://www.uniprot.org/uniprot/G1MEH7) | SILAVYSKDNY--------KRVTGTSLGGGTFLGLCC | L | LTGCETFEEALEMAAKGDSTNVDKLVKDIYG-G---DY |
| [sp\|G3WMT8#1](http://www.uniprot.org/uniprot/G3WMT8) | SILAVYSKDNY--------KRVTGTSLGGGTFLGLCC | L | LTGCETFEEALEMAAKGDSTNVDKLVKDIYG-G---DY |
| [sp\|UPI0002039D6B#1](http://www.uniprot.org/uniparc/UPI0002039D6B) | SILAVYSKDNY--------KRVTGTSLGGGTFLGLCC | L | LTGCETFEEALEMAAKGDSTNVDKLVKDIYG-G---DY |
| [sp\|B8A4W8#1](http://www.uniprot.org/uniprot/B8A4W8) | SILAVYSKDNY--------KRVTGTSLGGGTFLGLCC | L | LTGCSTFEEALAMATEGESTRVDKLVREIYG-G---DY |
| [sp\|UPI00022AF052#1](http://www.uniprot.org/uniparc/UPI00022AF052) | SILAVYSENNY--------KRVTGTSLGGGTFLGLCC | L | LTGCETFEEALEMASKGDSTNVDKLVKDIYG-G---DY |
| [sp\|UPI00017B31B4#1](http://www.uniprot.org/uniparc/UPI00017B31B4) | SILAVYSENNY--------KRVTGTSLGGGTFLGLCC | L | LTGCSTFDEALEMASQGESTRVDKLVRDIYG-G---DY |
| [sp\|UPI00022F3855#1](http://www.uniprot.org/uniparc/UPI00022F3855) | SILAVHSKDNY--------KRVTGTSLGGGTFLDLCS | L | LTGCESFEEALEMASKGDSTQADRLVRDIYG-G---DY |
| [sp\|UPI0001D55CC9#1](http://www.uniprot.org/uniparc/UPI0001D55CC9) | SILAVHSKDNXXXXXXXXSKHVFVCVLGGGTFLGLCS | L | LTGCESFEEALEMASKGDSTQADKLVRDIYG-G---DY |
| [sp\|UPI00016E45A6#1](http://www.uniprot.org/uniparc/UPI00016E45A6) | SILAVYSENNY--------KRVTGTSLGGGTFLGLCC | L | LTGCETFEEALEMASKGDSTNVDKLVKDIYG-G---DY |
| [sp\|UPI00016E12FF#1](http://www.uniprot.org/uniparc/UPI00016E12FF) | SILAVYSENNY--------KRVTGTSLGGGTFLGLCC | L | LTGCSTFDEALQMASQGESTRVDKLVRDIYG-G---DY |
| [sp\|G1KVX1#1](http://www.uniprot.org/uniprot/G1KVX1) | SILAVHSKDNY--------KRVTGTSLGGGTFLGLCS | L | LTGCESFEEALEMASKGDSTHADKLVRDIYG-G---DY |
| [sp\|F1Q3G1#1](http://www.uniprot.org/uniprot/F1Q3G1) | SILAVHSKDNY--------KRVTGTSLGGGTFLGLCC | L | LTGCESFEEALEMASKGDSTQADKLVRDIYG-G---DY |
| [sp\|G3H3U8#1](http://www.uniprot.org/uniprot/G3H3U8) | SILAVYSKDNY--------KRVTGTSLGGGTFLGLCC | L | LTGCETFEEALEMAAKGDSTNVDKLVKDIYG-G---DY |
| [sp\|Q4SDN2#1](http://www.uniprot.org/uniprot/Q4SDN2) | SILAVYSENNY--------KRVTGTSLGGGTFLGLCC | L | LTGCSTFDEALEMASQGESTRVDKLVRDIYG-G---DY |
| [sp\|A9ULG9#1](http://www.uniprot.org/uniprot/A9ULG9) | SILAVNSKDSY--------KRVCGTSLGGGTFLGLCS | L | LTGCESFEEALEMASAGDSTNADKLVRDIYG-G---DY |
| [sp\|F1QB40#1](http://www.uniprot.org/uniprot/F1QB40) | SILAVNSKDNY--------KRVTGTSLGGGTFLGLCC | L | LTGCETFEEALEMASKGDSTNVDKLVKDIYG-G---DY |
| [sp\|D2H9W3#1](http://www.uniprot.org/uniprot/D2H9W3) | SILAVHSKDNY--------KRVTGTSLGGGTFLGLCC | L | LTGCESFEEALEMASKGDSTQADKLVRDIYG-G---DY |
| [sp\|Q6DF85#1](http://www.uniprot.org/uniprot/Q6DF85) | SILAVQSKDSY--------KRVCGTSLGGGTFLGLCS | L | LTGCESFEEALEMASAGDSTNADKLVRDIYG-G---DY |
| [sp\|G1Q2F6#1](http://www.uniprot.org/uniprot/G1Q2F6) | SILAVHSKDNY--------KRVTGTSLGGGTFLGLCS | L | LTGCESFEEALEMASKGDSTHADKLVRDIYG-G---DY |
| [sp\|D2H6E1#1](http://www.uniprot.org/uniprot/D2H6E1) | SILAVYSKDNY--------KRVTGTSLGGGTFLGLCC | L | LTGCETFEEALEMAAKGDSTNVDKLVKDIYG-G---DY |
| [sp\|Q7SYD4#1](http://www.uniprot.org/uniprot/Q7SYD4) | SILAVNSKDNY--------KRVTGTSLGGGTFLGLCC | L | LTGCETFEEALEMASKGDSTNVDKLVKDIYG-G---DY |
| [sp\|D3ZKX7#1](http://www.uniprot.org/uniprot/D3ZKX7) | SILAVHSKDNY--------KRVTGTSLGGGTFLGLCS | L | LTGCESFEEALEMASKGDSTQADRLVRDIYG-G---DY |
| [sp\|Q4SQN8#1](http://www.uniprot.org/uniprot/Q4SQN8) | SILAVYSEDNY--------KRVTGTSLGGGTFLGLCC | L | LTGCETFEEALEMASKGDSTNVDKLVKDIYG-G---DY |
| [sp\|UPI00016E45A7#1](http://www.uniprot.org/uniparc/UPI00016E45A7) | SILAVYSENNY--------KRVTGTSLGGGTFLGLCC | L | LTGCETFEEALEMASKGDSTNVDKLVKDIYG-G---DY |
| [sp\|F6QRR1#1](http://www.uniprot.org/uniprot/F6QRR1) | SILAVHSKDNY--------KRVTGTSLGGGTFLGLCS | L | LTGCESFEEALEMASKGDSTHADKLVRDIYG-G---DY |
| [sp\|F7ECS7#1](http://www.uniprot.org/uniprot/F7ECS7) | SILAFGFTKQF--------FFFIFFSLGGGTFLGLCS | L | LTGCESFEEALEMASKGDSTQADKLVRDIYG-G---DY |
| [sp\|Q3UWJ1#1](http://www.uniprot.org/uniprot/Q3UWJ1) | SILAVHSKDNY--------KRVTGTSLGGGTFLGLCS | L | LTGCESFEEALEMASKGDSTQADRLVRDIYG-G---DY |
| [sp\|G3NMA3#1](http://www.uniprot.org/uniprot/G3NMA3) | SILAVYSETNY--------KRVTGTSLGGGTFLGLCC | L | LTGCSTFEEALEMASQGESTRVDKLVRDIYG-G---DY |
| [sp\|F6YKY4#1](http://www.uniprot.org/uniprot/F6YKY4) | SILAVYSKDNF--------KRVTGTSLGGGTFLGLCC | L | LTGCETFEEALEMAAKGDSTNVDKLVKDIYG-G---DY |
| [sp\|F6W6J8#1](http://www.uniprot.org/uniprot/F6W6J8) | SILAVHSKDNY--------KRVTGTSLGGGTFLGLCS | L | LTGCESFEEALEMASKGDSTQADKLVRDIYG-G---DY |
| [sp\|C3Y5V3#1](http://www.uniprot.org/uniprot/C3Y5V3) | SILAVYSQDDY--------KRISGTSLGGGTFLGLCC | L | LTGCDTFEDAIEMAANGNSTNVDKLVKDIYG-G---DY |
| [sp\|UPI0002038CCD#1](http://www.uniprot.org/uniparc/UPI0002038CCD) | SILAVHSKDNY--------KRVTGT------------ | - | -------------------------------------- |
| [sp\|UPI0001DE7E39#1](http://www.uniprot.org/uniparc/UPI0001DE7E39) | SILAVYSKDNY--------KRVTGT------------ | - | -------------------------------------- |
| [sp\|F6TKP2#1](http://www.uniprot.org/uniprot/F6TKP2) | SILAVYSKDNY--------KRVTGTSLGGGTFLGLCC | L | LTGCETFEEALEMAAKGDSTNVDKLVKDIYG-G---DY |
| [sp\|UPI00005A4E36#1](http://www.uniprot.org/uniparc/UPI00005A4E36) | SILAVYSKDNY--------KRVTGT------------ | - | -------------------------------------- |
| [sp\|Q8TE04-3#1](http://www.uniprot.org/uniprot/Q8TE04) | SILAVYSKDNY--------KRVTGT------------ | - | -------------------------------------- |
| [sp\|F6SYM8#1](http://www.uniprot.org/uniprot/F6SYM8) | SILAVYSKDNY--------KRVTGT------------ | - | -------------------------------------- |
| [sp\|UPI0001CBB0CB#1](http://www.uniprot.org/uniparc/UPI0001CBB0CB) | SILTVHSRHKF--------QRVSGTSLGGGTFLGLCC | L | LTGCQTFEEAIEMAASGDSTKVDKLVRDIYG-G---DY |
| [sp\|UPI00022B7852#1](http://www.uniprot.org/uniparc/UPI00022B7852) | SILAVYSKDNY--------KRV--------------- | - | -------------------------------------- |
| [sp\|B3KXQ7#1](http://www.uniprot.org/uniprot/B3KXQ7) | SILAVYSKDNY--------KRVTGTSLGGGTFLGLCC | L | LTGCETFEEALEMAAKGDSTNVDKLVKDIYG-G---DY |
| [sp\|F7EFE6#1](http://www.uniprot.org/uniprot/F7EFE6) | SILAVHSKD-----------------INEGTFLGLCT | L | LTGCESLEEALEMASKGDSTQADKL-------G---DY |
| [sp\|UPI00022C9F07#1](http://www.uniprot.org/uniparc/UPI00022C9F07) | SILAVYGSENY--------KRISGTSLGGGTFLGLCC | L | LTGCNTFEEAIELATGGDNTRVDKLVKDIYG-G---DY |
| [sp\|UPI00021A85E2#1](http://www.uniprot.org/uniparc/UPI00021A85E2) | SILAVYGPENY--------KRISGTSLGGGTFLGLCC | L | LTGCNTFEEAIELATGGDNTRVDKLVKDIYG-G---DY |
| [sp\|UPI0002063ECE#1](http://www.uniprot.org/uniparc/UPI0002063ECE) | SILAVYGPENY--------KRISGTSLGGGTFLGLCC | L | LTGCNTFEEAIELATGGDNTRVDKLVKDIYG-G---DY |
| [sp\|UPI00022471B0#1](http://www.uniprot.org/uniparc/UPI00022471B0) | SILTVQGPNDY--------KRISGTSLGGGTFLGLCC | L | LTGCNTFEEAIELATSGDSTKVDKLVKDIYG-G---DY |
| [sp\|E0VG17#1](http://www.uniprot.org/uniprot/E0VG17) | SMLTVYSPTDY--------KRVSGTSLGGGTFLGLCC | L | LTGCNSFEEAIDLATLGDHTRVDKLVRDIYG-G---DY |
| [sp\|E2AR36#1](http://www.uniprot.org/uniprot/E2AR36) | SILAVYGPENF--------KRISGTSLGGGTFLGLCC | L | LTGCNTFEEAIELATGGDNTKVDKLVKDIYG-G---DY |
| [sp\|E2C1K4#1](http://www.uniprot.org/uniprot/E2C1K4) | SILTVYGPEDY--------KRISGTSLGGGTFLGLCC | L | LTGCNTFEEAIELATGGDNTKVDKLVKDIYG-G---DY |
| [sp\|F1P3P8#1](http://www.uniprot.org/uniprot/F1P3P8) | SILAVYSKENY--------RWVTGTSLGGGTFFGLCC | L | LTGCSTFEEAMDMASHGDSTKVDKLVRDIYG-G---DY |
| [sp\|E9IWQ0#1](http://www.uniprot.org/uniprot/E9IWQ0) | SILAVYGPENY--------KRISGTSLGGGTFLGLCC | L | LTGCNTFEEAIGLATGGDNTRVDKLVKDIYG-G---DY |
| [sp\|D6WCF9#1](http://www.uniprot.org/uniprot/D6WCF9) | SVLAVYSATDY--------KRISGTSLGGGTFLGLCC | L | LTGCNTFEEAIQLAAEGDNTRVDKLVRDIYG-G---DY |
| [sp\|UPI0001C0B3A4#1](http://www.uniprot.org/uniprot/UPI0001C0B3A4) | SVLAVYSATDY--------KRISGTSLGGGTFLGLCC | L | LTGCNTFEEAIQLAAEGDNTRVDKLVRDIYG-G---DY |
| [sp\|UPI0001C0B3A3#1](http://www.uniprot.org/uniprot/UPI0001C0B3A3) | SVLAVYSATDY--------KRISGTSLGGGTFLGLCC | L | LTGCNTFEEAIQLAAEGDNTRVDKLVRDIYG-G---DY |
| [sp\|D3TLZ6#1](http://www.uniprot.org/uniprot/D3TLZ6) | SILAVYGPDNY--------KRVSGTSLGGGTFLGLCC | L | LTGCNTFEEAIQLATKGDNRKVDKLVRDIYG-G---DY |
| [sp\|E3X8B3#1](http://www.uniprot.org/uniprot/E3X8B3) | SVLAVRGPDNY--------KRISGTSLGGGTFLGLCC | L | LTGCDTFEEAIQLATKGDHKKVDKLVKDIYG-G---DY |
| [sp\|Q7PVC2#1](http://www.uniprot.org/uniprot/Q7PVC2) | SVLAVRGPDNY--------KRISGTSLGGGTFLGLCC | L | LTGCETFEEAIQLATKGDHKKVDKLVKDIYG-G---DY |
| [sp\|UPI0000524C29#1](http://www.uniprot.org/uniparc/UPI0000524C29) | SIMLVRSQEDY--------ARVSGTSLGGGTFLGLCC | L | LTGCETYDEAIDLATKGDSTKVDKLVSDIYGEG---GY |
| [sp\|B4MLJ4#1](http://www.uniprot.org/uniprot/B4MLJ4) | SILAVYGPDNY--------KRISGTSLGGGTFLGLCC | L | LTGCTTFEEAIQLATKGDNRKVDKLVKDIYG-G---DY |
| [sp\|F6X5M5#1](http://www.uniprot.org/uniprot/F6X5M5) | SIMLVRSQEDY--------ARVSGTSLGGGTFLGLCC | L | LTGCETYDEAIDLATKGDSTKVDKLVSDIYGEG---GY |
| [sp\|B4HBV4#1](http://www.uniprot.org/uniprot/B4HBV4) | SILAVYGPDNY--------KRISGTSLGGGTFLGLCC | L | LTGCTTFEEAIQLATKGDNRKVDKLVKDIYG-G---DY |
| [sp\|Q2LYJ4#1](http://www.uniprot.org/uniprot/Q2LYJ4) | SILAVYGPDNY--------KRISGTSLGGGTFLGLCC | L | LTGCTTFEEAIQLATKGDNRKVDKLVKDIYG-G---DY |
| [sp\|Q9VPG4#1](http://www.uniprot.org/uniprot/Q9VPG4) | SILAVYGPDNY--------KRISGTSLGGGTFLGLCC | L | LTGCTSFEEAIQLATKGDNRKVDKLVKDIYG-G---DY |
| [sp\|Q9VPG5#1](http://www.uniprot.org/uniprot/Q9VPG5) | SILAVYGPDNY--------KRISGTSLGGGTFLGLCC | L | LTGCTSFEEAIQLATKGDNRKVDKLVKDIYG-G---DY |
| [sp\|B4QRY3#1](http://www.uniprot.org/uniprot/B4QRY3) | SILAVYGPDNY--------KRISGTSLGGGTFLGLCC | L | LTGCTSFEEAIQLATKGDNRKVDKLVKDIYG-G---DY |
| [sp\|B4PF95#1](http://www.uniprot.org/uniprot/B4PF95) | SILAVYGPDNY--------KRISGTSLGGGTFLGLCC | L | LTGCTSFEEAIQLATKGDNRKVDKLVKDIYG-G---DY |
| [sp\|B4PF92#1](http://www.uniprot.org/uniprot/B4PF92) | SILAVYGPDNY--------KRISGTSLGGGTFLGLCC | L | LTGCTSFEEAIQLATKGDNRKVDKLVKDIYG-G---DY |
| [sp\|Q9NHN2#1](http://www.uniprot.org/uniprot/Q9NHN2) | SILAVYGPDNY--------KRISGTSLGGGTFLGLCC | L | LTGCTSFEEAIQLATKGDNRKVDKLVKDIYG-G---DY |
| [sp\|B4IA96#1](http://www.uniprot.org/uniprot/B4IA96) | SILAVYGPDNY--------KRISGTSLGGGTFLGLCC | L | LTGCTSFEEAIQLATKGDNRKVDKLVKDIYG-G---DY |
| [sp\|B3NIK9#1](http://www.uniprot.org/uniprot/B3NIK9) | SILAVYGPDNY--------KRISGTSLGGGTFLGLCC | L | LTGCTSFEEAIQLATKGDNRKVDKLVKDIYG-G---DY |
| [sp\|B3M986#1](http://www.uniprot.org/uniprot/B3M986) | SILAVYGPDNY--------KRISGTSLGGGTFLGLCC | L | LTGCTSFEEAIQLATKGDNRKVDKLVKDIYG-G---DY |
| [sp\|B7PXJ2#1](http://www.uniprot.org/uniprot/B7PXJ2) | SILAVHSPTQY--------HRVTGTSLGGGTFLGLCC | L | LTGCETFEEAIDLAQRGDSTHVDKLVRDIYG-G---DY |
| [sp\|B4KYY9#1](http://www.uniprot.org/uniprot/B4KYY9) | SILAVYGPDNY--------KRISGTSLGGGTFLGLCC | L | LTGCTTFEEAIQLATKGDNRKVDKLVKDIYG-G---DY |
| [sp\|B4LD66#1](http://www.uniprot.org/uniprot/B4LD66) | SILAVYGPDNY--------KRISGTSLGGGTFLGLCC | L | LTGCTTFEEAIQLATKGDNRKVDKLVKDIYG-G---DY |
| [sp\|Q16M65#1](http://www.uniprot.org/uniprot/Q16M65) | SVLAVRGPDNY--------KRISGTSLGGGTFLGLCC | L | LTGCTTFEEAIQLATKGDHTKVDKLVKDIYG-G---DY |
| [sp\|UPI0000586B1A#1](http://www.uniprot.org/uniparc/UPI0000586B1A) | SMLAVRSHNDF--------ERVTGTSLGGGTFLGLCC | L | LTGCETFEEALDMASKGDNTRADKLVRDIYG-G---DY |
| [sp\|E9FSL9#1](http://www.uniprot.org/uniprot/E9FSL9) | SILAVYGPNNF--------KRVTGTR----------- | - | ---CQSYEEAIALAAAGDSTKVDKKVRDIYG-G---DY |
| [sp\|B4IYF2#1](http://www.uniprot.org/uniprot/B4IYF2) | SILAVYGPDNY--------KRISGTSLGGGTFLGLCC | L | LTGCTTFEEAIQLATKGDNRKVDKLVKDIYG-G---DY |
| [sp\|A7SEX1#1](http://www.uniprot.org/uniprot/A7SEX1) | SIMAVYSAEKF--------DRISGTSLGGGTFLGLCC | L | MTGCTTFEEALSMAARGDSTKVDKLVKDIYG-G---DY |
| [sp\|B0WFV1#1](http://www.uniprot.org/uniprot/B0WFV1) | SVLAVRGPDNY--------KRISGTSLGGGTFLGLCC | L | LTGCETFEEAIQLATRGDHKRVDKLVKDIYG-G---DY |
| [sp\|UPI000206194C#1](http://www.uniprot.org/uniparc/UPI000206194C) | SVLAVYGPNNY--------KRISGTSIGGGTFLGLCS | L | LTGCNTFDEAIELAANGDNVKVDKLVRDIYG-G---SY |
| [sp\|B3SB80#1](http://www.uniprot.org/uniprot/B3SB80) | SILAVQSPDEY--------YRVTGSSIGGGTFLGLCC | L | LTGCETFEEALRMAENGDSTKVDKLVRDIYG-G---SY |
| [sp\|E5SCW7#1](http://www.uniprot.org/uniprot/E5SCW7) | SILAVAEDHSF--------RRVSGTSLGGGTFHGLCC | L | LTGCETFEQALELASLGENNKVDKLVGDIYG-G---DY |
| [sp\|F1KZL9#1](http://www.uniprot.org/uniprot/F1KZL9) | SVLAVRGHDDF--------ERISGSSIGGGFFQGLCA | V | MCGCETFEEAIELASRGDNRNVDKLVKDIYG-T---GY |
| [sp\|Q6IDG4#1](http://www.uniprot.org/uniprot/Q6IDG4) | SILAVYGPDNY--------KRISGTSLGGGTFLGLCC | L | LTGCTSFEEAIQLATKGDNRKVDKLVKDIYG-G---DY |
| [sp\|F7DF56#1](http://www.uniprot.org/uniprot/F7DF56) | SILAVYSKDNY--------RRV--------------- | - | -------------------------------------- |
| [sp\|UPI000223EBB3#1](http://www.uniprot.org/uniparc/UPI000223EBB3) | SILAVYSKDNY--------RRV--------------- | - | -------------------------------------- |
| [sp\|A8Q0S4#1](http://www.uniprot.org/uniprot/A8Q0S4) | SVLAVRGHNQF--------KRIGGSSIGGGFFQGICA | I | MCGCETFEEAIELASRGDNKNVDKLVKDIYG-S---GY |
| [sp\|UPI00021A4115#1](http://www.uniprot.org/uniparc/UPI00021A4115) | SILHVTGPSQF--------KRIGGTSVGGGTFLGLCS | L | LTGCDNFQDAITLAEKGDHTKVDKMVRDIYG-G---DY |
| [sp\|UPI0002035CE0#1](http://www.uniprot.org/uniparc/UPI0002035CE0) | SILAVYSKDNY--------KRV--------------- | - | -------------------------------------- |
| [sp\|E1FZ15#1](http://www.uniprot.org/uniprot/E1FZ15) | SVLAVRGHNQF--------ERIGGSSIGGGFFQGLCA | I | MCGCETFEEAIELASRGDNKNVDKLVKDIYG-S---GY |
| [sp\|F6ZYC4#1](http://www.uniprot.org/uniprot/F6ZYC4) | YVLFVFPQE--------------STLCNTSTFLGLCC | L | LTGCETYDEAIDLATKGDSTKVDKLVSDIYGEG---GY |
| [sp\|E9QCQ7#1](http://www.uniprot.org/uniprot/E9QCQ7) | SILAV-------------------------------- | - | -------------------------------------- |
| [sp\|Q5RFL6#1](http://www.uniprot.org/uniprot/Q5RFL6) | SILAVYSKDNY--------KRV--------------- | - | -------------------------------------- |
| [sp\|F2U7V3#1](http://www.uniprot.org/uniprot/F2U7V3) | SILKVNEDGQN--------ERVGGSSLGGATFFGLAS | L | LTGCKSFDEALALAGEGEASGVDLLVGDIYG-G---DY |
| [sp\|G5AFB3#1](http://www.uniprot.org/uniprot/G5AFB3) | SVLYVKAPGDY--------ERVSGSSIGGGTYWGLCR | L | MTHCESYDEALDLCVHGTNKTVDMSVGDIYG-G---AY |
| [sp\|D0N1V9#1](http://www.uniprot.org/uniprot/D0N1V9) | SVLYVKGPGDY--------ERVSGSSIGGGTYWGLCR | L | MTNCDTYDEALDLCVHGSNKSVDMSVGDIYG-G---AY |
| [sp\|A9V0H1#1](http://www.uniprot.org/uniprot/A9V0H1) | SILKVHADGRH--------QRVGGTAIGGATFYGLCA | A | LTSCDSFDAALQLAAQGDASRVDLLVGDIYG-G---DY |
| [sp\|D0NIF5#1](http://www.uniprot.org/uniprot/D0NIF5) | SILKITGESQY--------ERVSGTSLGGGTFLGLCR | A | LSKLHTFDEAMDASVEGDSNEVDMTVGDIYGTA---GY |
| [sp\|D8LL03#1](http://www.uniprot.org/uniprot/D8LL03) | SIIKVDGPAKF--------KRVSGSSIGGGTYWGLCR | L | LTEATSYDESLDMARQGNSDKVDMLVGDIYG-R---GY |
| [sp\|UPI00005A441E#1](http://www.uniprot.org/uniparc/UPI00005A441E) | ------------------------------------- | - | -------------------------------------- |
| [sp\|F0WP99#1](http://www.uniprot.org/uniprot/F0WP99) | SILHVKGPEDF--------DRVSGSSIGGGTYWGLCR | L | LTKCKSYDEALDVCVHGTNSTVDMSVGDIYG-G---AY |
| [sp\|B7G788#1](http://www.uniprot.org/uniprot/B7G788) | SILRVDGPRKH--------ERVSGSTIGGGTYWGLIR | L | LTDIDDFEHVMRLAEQGDATKVDMMVGDIYG-NKSDAL |
| [sp\|UPI000194E9EB#1](http://www.uniprot.org/uniparc/UPI000194E9EB) | SILAVYSKDNY--------KRVTGTSLGGGTFFGLCC | L | LTGCSTFEEALEMASHGDSTKVDKLVRDIYG-G---DY |
| [sp\|D3ZEV6#1](http://www.uniprot.org/uniprot/D3ZEV6) | ------------------------------------- | - | -------------------------------------- |
| [sp\|UPI00005A5E52#1](http://www.uniprot.org/uniparc/UPI00005A5E52) | SILAVYSKDNY--------KRV--------------- | - | -------------------------------------- |
| [sp\|D8MBD9#1](http://www.uniprot.org/uniprot/D8MBD9) | SILKVTSENVC--------ERVGGCPMGGGTFFGLAR | L | LAGGLSFDEALKQAGEGNKSNVDMLVRDIYG-G---NY |
| [sp\|D0P0J2#1](http://www.uniprot.org/uniprot/D0P0J2) | SILKITGESQY--------ERVSGTSLGGGTFLGLCR | A | LFKLHTFDEAMDASVEGDSNEVDMTVGDIYGTA---GY |
| [sp\|B5YN27#1](http://www.uniprot.org/uniprot/B5YN27) | SILRVDGPRQH--------ERVSGSTIGGGTYWGLCR | L | LTGSDSFSDVLNLAMKGDPSKVDMMVGDIYG-KDSNAL |
| [sp\|E5RHA5#1](http://www.uniprot.org/uniprot/E5RHA5) | ------------------------------------- | - | -------------------------------------- |
| [sp\|A9RE48#1](http://www.uniprot.org/uniprot/A9RE48) | SILRVDAAERF--------ERVGGTSLGGSTFLGLTS | A | LTNCTTFEEAINLAREGDSTQIDMLVGDIYG-G---DY |
| [sp\|Q6XHL7#1](http://www.uniprot.org/uniprot/Q6XHL7) | ------------------------------------- | - | -----------QLATKGDNRKVDKLVKDIYG-G---DY |
| [sp\|UPI0002035CE0#2](http://www.uniprot.org/uniparc/UPI0002035CE0) | ------------------------------------- | - | -------------------------------------- |
| [sp\|G5E2N0#1](http://www.uniprot.org/uniprot/G5E2N0) | SILAVHSKDCY--------KRVCGTSLGGGTFLGLCS | L | LTGCESFEEALEM------------------------- |
| [sp\|F4WR35#1](http://www.uniprot.org/uniprot/F4WR35) | ------------------------------------- | - | -------------------------------------- |
| [sp\|UPI0000588FE0#1](http://www.uniprot.org/uniparc/UPI0000588FE0) | SMLAVRSHNDF--------ER---------------- | - | -------------------------------------- |
